# Continuous Inking Affects the Biological and Biochemical Responses of Cuttlefish *Sepia pharaonis*

**DOI:** 10.3389/fphys.2019.01429

**Published:** 2019-11-29

**Authors:** Maowang Jiang, Chenxi Zhao, Runxuan Yan, Jianping Li, Weiwei Song, Ruibing Peng, Qingxi Han, Xiamin Jiang

**Affiliations:** School of Marine Sciences, Ningbo University, Ningbo, China

**Keywords:** inking, predator–prey interactions, biochemical responses, animal care and welfare, *Sepia pharaonis*

## Abstract

Several marine mollusks, including cephalopods (cuttlefish, squid, and octopus) and gastropods (e.g., sea hares), can release a colored ink secretion when chased by predators or stimulated. Ink release is part of a defensive response, but the threshold for the biochemical responses caused by stimulation is unknown. The present study aimed to reveal antipredator responses of cuttlefish, such as escaping via inking and/or jetting, and to investigate its biological and biochemical responses to continuous ink release. Results showed that the behavioral responses to continuous ink release mainly manifested as blazing body pattern changes. Cuttlefish escaped from predators covered by jetting/inking and warned the potential threats by displaying a unique body pattern. Moreover, persistent inking in the presence of an overt stimulus caused uncontrollable ink release from the ink duct/anal canal (loss of control). This study first verified the characteristics of the cuttlefish ink solution, prepared a standard curve of ink solution concentrations, and fitted the relationship function between the release frequency and the released ink weight. Biological statistics indicated that cuttlefish has the ability to continuously release ink (releasing ∼90% of the ink from the ink sac) and that the individuals adapted well during the recovery period. However, re-releasing ink would result in “overexploitation” and high mortality. Hexokinase (HK), pyruvate kinase (PK), and superoxide dismutase (SOD) activities, as well as malondialdehyde (MDA) concentration increased or remained stable in different tissues after releasing ink. The expression of heat shock protein 90 and arginine kinase (AK) were upregulated by stimuli in all tissues. Biochemical changes indicated that continuous inking not only consumed considerable energy but also damaged the tissues. In summary, cuttlefish released almost 90% of their ink for active defense against predators, and it took ∼30 days for the ink sac to be refilled, but “overexploitation” resulted in serious physiological damage. These findings will be helpful to further study the defense and ink release mechanisms and to consider animal health and welfare when using cephalopods as experimental animals and for aquaculture practices.

## Introduction

Predator–prey interactions can exert strong selection pressure that affects the evolution of antipredation defense measures (Paul and Pennings, 1991; Zimmer and Ferrer, 2007; Hay, 2009). These defenses include behavioral adaptations (crypsis), body coloration (camouflage and threats), mechanical defenses (fleeing), and chemical defenses (electrical, poison, and colored secretion) (Pawlik, 1993; Hanlon and Messenger, 1996; Wyatt, 2003; Murphy, 2006; Glaudas and Winne, 2007). Prey species that may encounter various predators must be adapted for protection against various predation methods and must have defenses that affect organisms with very different sensory systems and adaptations of their own. Many marine mollusks release secretions as defense against predators, e.g., cephalopods (cuttlefish, squid, and octopus) and gastropods (sea hares) release a colored ink secretion (Hanlon and Messenger, 1996; Kasugai, 2001; Caldwell, 2005; Wood et al., 2010; Staudinger et al., 2011).

Cephalopods are prey to numerous marine vertebrates, including mammals, fish, and diving seabirds (Packard, 1972; Croxall and Prince, 1996; Smale, 1996) because they are soft bodied and lack hard protective structures such as spines and shells (Packard, 1972; Hanlon and Messenger, 1996). As an example of the high exposure of cephalopods to predators, one study of the Caribbean reef squid reported an average of seven encounters per hour (Hanlon and Messenger, 1996). Cephalopods have several types of defenses against predators similar to other species, including the ability to change color, shape, and texture, which can provide camouflage and crypsis that confuse, threaten, frighten, or bluff predators (Hanlon and Messenger, 1996; King and Adamo, 2006; Hanlon, 2007; Bush et al., 2009). Cephalopod ink is typically thought to have a defensive function through its visual effects on predators. Inking involves the release of a mass of black chemicals that can take different forms, i.e., a diffuse plume, a gelatinous mass known as a pseudomorph, or other forms and shapes (Shimek, 1983; Huffard and Caldwell, 2002; Bush and Robison, 2007).

Direct interactions with predators are often unavoidable, and inking is a visual stimulus that may act as an important defense in the emergence of danger. Cohesive ink is released as a decoy supposed to attract predators, while diffuse ink is released as a smokescreen covering the retreat (Humphries and Driver, 1970; Driver and Humphries, 1988). Sea hare ink has an adverse effect on the chemical sensory organs of predators, acting as a repulsive substance that prevents predators from attacking, an aversive substance that causes the predator to reject an inking animal once it has been taken into the mouth of the predator, or as a substance that disrupts the senses of the predator’s sensory system and thus affects the predator’s ability to assess or consume the animal (Sheybani et al., 2009; Nusnbaum and Derby, 2010; Wood et al., 2010). Furthermore, cephalopods also produce protean behaviors, which include unpredictable erratic escape behaviors such as jetting and inking (Hanlon and Messenger, 1996).

Particularly, changes in cephalopod behavior against predators suggest that their reaction to jetting/inking has been underestimated. Encountering a predator is a common challenge for a prey animal, and it is important to assess the biochemical changes and consequent energy stress (Sato et al., 2016). Cuttlefish releases ink from the ink sac when subjected to external stimuli (e.g., pressure) (Nithya et al., 2011). To be well prepared for danger, the ink gland cells keep producing melanin to the ink sac, the typical effector organ delegated to storage after release a large amount of ink (Palumbo et al., 2000). We observed that the pigmentation stage of embryonic development is capable of releasing ink when manipulated vigorously. Besides, cuttlefish release ink during rearing, e.g., weaning, competition for food, mating, and rapid changes in temperature and salinity (Le et al., 2014; Lee et al., 2016; Jiang et al., 2018). Furthermore, overrelease of ink results in the individual’s death. However, cuttlefish are always encountering predators accompanied by inking and/or jetting in nature, but the ability of continuously releasing ink, the effect of the inking behavior on the cuttlefish itself, and the ink synthesis rate are still unknown. We investigated the effects of continuous ink release on survival, behavior, and biochemical characteristics of cuttlefish *Sepia pharaonis* and tried to elucidate the biological and biochemical responses of cephalopods to ink release, which might provide a basis for further research.

## Materials and Methods

### Animals and Rearing Conditions

*Sepia pharaonis* eggs belonged to the second generation (F_2_) of cuttlefish reared at our research facility. Experiments were conducted at the Lai Fa Aquaculture Co., Ltd. (29° 59′ N, 121° 99′ E) (Zhejiang Province, China), which specializes in aquatic technology research and application development. Posthatching, cuttlefish were reared in a cement pond (7.8 m × 3.8 m × 1.6 m, length × width × depth; area, 30 m^2^), as described for the species by Jiang et al. (2018). In short, newly hatched juveniles were fed enriched live rotifers (*Artemia nauplii*) the first 3 days posthatching and then live mysids (*Hyperacanthomysis brevirostris*) twice a day (8:00 and 16:00) *ad libitum*.

After three treatments causing ink release events, the cuttlefish were cultured in the cement pond and fed frozen shrimp (white shrimp *Penaeus vannamei*) twice a day (at 7:00 and 16:00), ensuring that the cuttlefish had sufficient food supply (20% of the cuttlefish weight) at each feeding time (Domingues et al., 2004). Gentle aeration provided by air stone and airlift for the cement pool (1–1.5 m^2^/piece). Natural seawater was filtered by a filter bed and ultraviolet sterilizers before it was pumped into the tank. The water quality parameters were as follows: salinity at 29 ± 0.6‰, temperature at 26 ± 0.9°C, pH 8.55 ± 0.42, ammonia nitrogen controlled at 0.05 ± 0.03 mg L^–1^, and dissolved oxygen at 6.63 ± 0.21 mg L^–1^. Temperature, salinity, and dissolved oxygen were measured daily with a YSI Pro DSS (YSI)^1^, and seawater was 30% refreshed every day under the same conditions. Using natural light as a light source, a day–night cycle (12 h:12 h) was maintained during the experiment with an intensity of 200 lx. Light intensity was detected using a handheld illuminometer (Sanwa, LX2). Low light intensity was adopted to maintain low-stress levels (Koueta and Boucaud, 2003; Sykes et al., 2003).

### Experimental Procedure and Estimation of the Number of Animals

The total number of animals used to determine the average quantity of ink released by cuttlefish. Animals of adequate age and size (50 days old; dorsal mantle length, 5.0 ± 0.3 cm; body weight, 20.12 ± 2.65 g), and similar in size to the ones that are expected to be used during a single inking event. In addition, the weight of released ink decreased significantly with the increase in ink release frequency, the final number of individuals (dorsal length, 5.5 ± 0.3 cm; body weight, 25.36 ± 3.82 g) to be utilized in the present study resulted to be larger than 350.

At the cuttlefish, they were forced to release ink every 24 h in three ink release events (days 1-3). Each individual was placed in a foam box (58 cm × 34 cm × 26 cm, length × width × depth) containing 20 L of seawater and tested one at a time. A string net (10 cm in diameter) was placed in the foam box and simulate an attack on the cuttlefish by a predator. This resulted in a defensive behavior response to escaping and inking and/or jetting and burst swimming backward. The string net was placed directly in front of the cuttlefish while retreating for 30–60 s until it stopped swimming and showed no obvious reaction to the stimulus. The cuttlefish ink solution in the foam box was then stirred with a hand-held stirrer (GTH-100; ∼2 min), and the concentration of the cuttlefish ink was measured. This allowed to calculate the weight of the released ink released. Each time the animal was dislocated from the foam box, it was temporarily housed in a plastic basket (55 cm × 18 cm, diameter × height). After three ink release events, the cuttlefish had to release ink by the same method after 5, 10, 15, 20, 25, and 30 (housing in the cement pond) and stimulated to obtain the ink after each period (*n* = 50) as well as to allow for the development of the ink after the continuous stimulation and the effect of re-releasing ink on the cuttlefish itself.

The ink sac was collected and weighed by dissecting the cuttlefish that had been released (dorsal mantle length, 5.4 ± 0.7 cm; weight, 23.28 ± 6.75 g, *n* = 50). Ink weights from the three release events were added to calculate total weight (TRW, mg) and ink weight in the ink sac (IWIS, mg) and were analyzed by descriptive statistics.

### Characteristics of the Cuttlefish Ink Solution and the Concentration Standard Curve Preparation

#### Characteristics of the Cuttlefish Ink Solution

**FIGURE 1:**
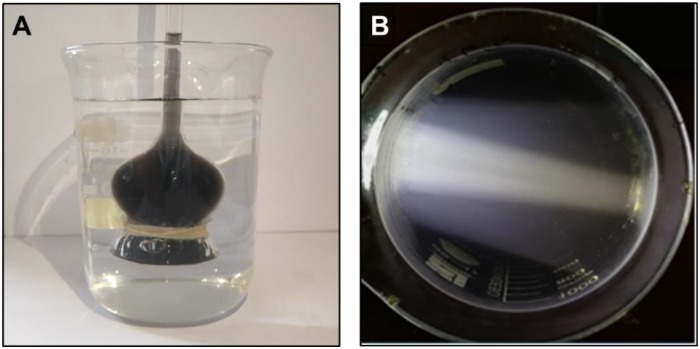
Characteristics of cuttlefish ink solution. **(A)** The ink solution was added to the long-neck funnel with a semipermeable membrane. **(B)** The beaker containing ink solution was illuminated with a light placed parallel to the side of the beaker.

The fresh cuttlefish ink was diluted to a certain concentration and made uniform by stirring using a magnetic stirrer. No aggregation or sedimentation occurred at room temperature (30 ± 0.8°C) for 3 days, indicating that the cuttlefish ink solution is homogeneous and stable in a short period. The ink solution was put into the long neck funnel with a semipermeable membrane at the bottom and then transferred to the beaker full of deionized water, as shown in Figure 1A. A light was placed parallel to the side of the beaker to illuminate the ink solution, and a clear light path was observed (Figure 1B).

#### Preparation of the Concentration Standard Curve

**FIGURE 2:**
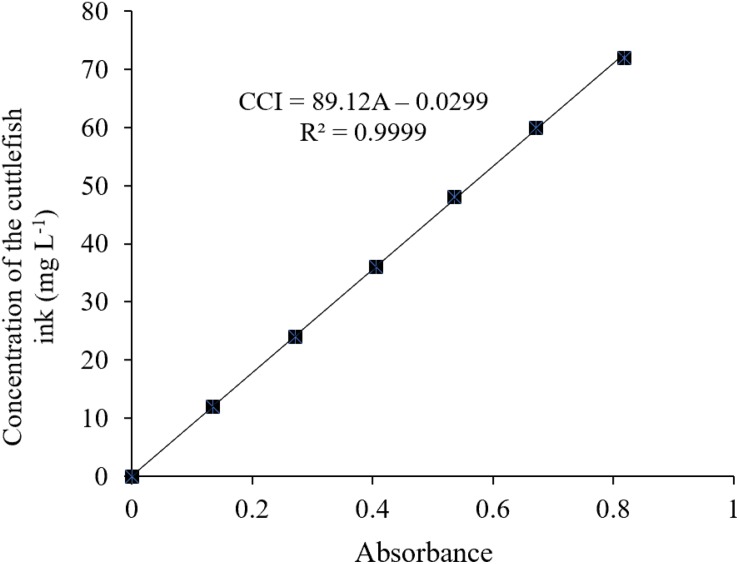
Standard curve of ink concentration of *Sepia pharaonis*.

1. A total of 300 mg fresh cuttlefish ink was added to a 1.5-L beaker and diluted with 1,000 mL deionized water and stirred using a magnetic stirrer (5 min) to prepare a cuttlefish ink solution of 300 mg L^–1^.
2. According to the national standard method (Editorial Board of the State Environmental Protection Administration (SEPA), 2002), 0, 2, 4, 6, 8, 10, and 12 mL of the above 300 mg L^–1^ cuttlefish ink solution were added to separate 50-ml volumetric flasks. Deionized water was added to a volume of 50 mL and was shook well to get ink solutions of 0, 12, 24, 24, 36, 48, 60, and 72 mg L^–1^, respectively.
3. The specific wavelength of the cuttlefish ink solution was 320 nm, determined using an ultraviolet–visible spectrophotometer (UNIC 2800 UV/VIS) at full wavelength scanning. The deionized water was used as a reference, and the absorbance values of the above-diluted solutions at 320 nm were 0, 0.135, 0.271, 0.406, 0.536, 0.671, and 0.819. The standard curve was drawn with absorbance (OD) as the *X*-axis and the concentration of the cuttlefish solution as the *Y* coordinate (Figure 2).

### Behavior

Cuttlefish behavior was studied using video cameras (SONY HDR-CX450) during inking. Four behavioral elements were used to assess the effect of release ink on the cuttlefish. (1) Jet frequency (*n*) was calculated as the number of jets (including ink and water jetting) during a single inking treatment. Direct observations were also performed at the same time as the video recordings to count the jet frequency. (2) Body pattern changes included chromatic changes and textural, postural, and locomotor components. Cephalopods such as cuttlefishes, octopods, and squids can change their body color and postural behaviors rapidly and also exhibit a variety of visually complex appearances (Holmes, 1940; Nakajima and Ikeda, 2017). These appearances comprise a combination of chromatic, textural, postural, and locomotor components for both camouflage and communication (Neill, 1971; Hanlon and Messenger, 1988). Body pattern changes will provide a useful foundation for quantitative behavioral analyses. (3) Ink solution properties were revealed, including ink forms, mucus volume, and dispersing changes in water. Inking involves ejecting a mass of black substances that take different forms. Ink forms change with continuous release, and defensive effect can be used as an indicator to study the characteristics of the ink secretion. (4) Swimming and feeding activities were also studied. Continuous ink release consumes considerable energy, and how inking affects the ability of a cuttlefish to escape from predators and food intake can be used as an effective parameter to assess the impact of inking on the activity and feeding of the organism.

### Tissue Sampling and Enzyme Activity

#### Tissue Sampling

As a control group, 10 cuttlefish juveniles (dorsal mantle length, 5.4 ± 0.2 cm; weight, 24.79 ± 3.48 g) were anesthetized with 5% alcohol before inking treatment. Samples of liver, gill, muscle, and brain tissues were immediately frozen in liquid nitrogen and stored at –80°C. The test group was sampled on the first (day 1), second (day 2), third (day 3), and fourth (day 8) inking treatments. Six cuttlefish juveniles were anesthetized with 5% ethanol for each treatment group, and tissues were sampled and stored as described above.

#### Enzyme Activity

The activities of hexokinase (HK), pyruvate kinase (PK), superoxide dismutase (SOD), and malondialdehyde (MDA) were measured with commercial assay kits (Nanjing Jiancheng Bioengineering Institute, Nanjing, China)^2^ following the manufacturer’s instructions. The assays are briefly described below.

The activities of HK (EC 2.7.1.1) and PK (EC 2.7.1.40) were determined following the procedures described by Tranulis et al. (1996) and Foster and Moon (1985), respectively. The absorbance of the samples was read at 340 nm. The enzyme activities were expressed as per milligram of total protein (specific activity). The total protein content in crude extracts was determined at 30°C using bovine serum albumin as a standard based on the Bradford (1976) method.

The activities of SOD (WST-1 method) and MDA (thiobarbituric acid method) concentration were measured at 450 and 532 nm using the xanthine oxidase and thiobarbituric acid methods according to Wang and Chen (2005) and Placer et al. (1966), respectively. The levels are expressed as units of SOD per milligram protein and nanomole of MDA per milligram protein.

### Real-Time PCR Analysis of Heat Shock Protein 90 and Arginine Kinase Gene Expression Patterns

#### RNA Extraction and First-Strand cDNA Synthesis

Total genomic RNA was extracted from tissue (liver, gill, and, muscle) samples of juveniles *S. pharaonis* using a Trizol RNA extraction reagent (Invitrogen, Carlsbad, CA, United States), following the method of Rio et al. (2010). The concentration of RNA was measured using Nanodrop ND-1000 (Thermo Scientific, United States). Electrophoresis in 1.0% formaldehyde-denaturing agarose gel was used for assessing the quality of the RNA, and the purity of RNA was checked by measuring the ratio of OD260/OD280 (1.8–2.0). For the synthesis of first-strand complementary DNA (cDNA), 1 μg of RNA was treated with 1 U of DNase I (Sigma-Aldrich, United States). This step helps to avoid DNA contamination. The cDNA was synthesized from 1 μg of total RNA using real-time PCR kit (Takara, Japan) following the instructions for SYBR^®^ PrimeScript^TM^. β-Actin, the constitutively expressed housekeeping gene was used for sample normalization and as positive control. The PCR amplification of β-actin was carried out for the confirmation of cDNA synthesis.

#### Real-Time PCR Analysis

The fragment sequence of the heat shock protein 90 (HSP90) gene, in this experiment, was derived from the database of the *S. pharaonis* transcription constructed by this group. Arginine kinase (AK) primers were designed from this group (Si et al., 2016a, b). All primer pairs (Table 1) for real-time PCR were synthesized by Shanghai Sangon Biotech Co., Ltd. (Shanghai, China). The fluorescent quantitative PCR reaction solution consisted of 12.5 μL SYBR^®^ premix Ex Taq^TM^ (2×), 0.5 μL PCR forward primer (10 μM), 0.5 μL PCR reverse primer (10 μM), 2.0 μL RT reaction mix (cDNA solution), and 9.5 μL dH_2_O. All samples were analyzed in triplicate wells using the following cycling parameters: 94°C for 5 min, followed by 40 cycles consisting of 94°C for 5 s, 60°C for 10 s, and 72°C for 15 s. The fluorescent flux was then recorded, and the reaction was continued at 72°C for 3 min. We measured the dissolution rate between 65 and 92°C. Every increase of 0.2°C was maintained for 1 s, and the fluorescent flux was recorded simultaneously. The efficiencies close to 100% were used in the 2^–ΔΔCT^ method for relative gene expression calculation (Livak and Schmittgen, 2001). We measured the PCR efficiency by constructing a standard curve using a serial dilution of cDNA; ΔΔ*C*_T_ = (*C*_T, Target_ - *C*_T, β –actin_) time *x* - (*C*_T, Target_ - *C*_T, β –actin_) time 0.

**TABLE 1:** Target genes and sequences of primers used to investigate messenger RNA (mRNA) levels.

**Target gene**	**Primer**	**Primer sequence (5′-3′)**
β-actin	Fw	TCCTGACCGAGAGAGGCTAC
	Rv	CTGCTCGAAGTCAAGAGCCA
HSP90	Fw	TCGAACTCATCCCCGATCTGA
	Rw	CTTTGTGCCGGATTTAGCGAT
AK	Fw	TTGCTGAAGTCCTTGATGCYGT
	Rw	TCATGGTRGTACCCAAGTTGC

### Statistical Analysis

The results were presented as the means ± SD (*n* = 3). All data were subjected to one-way ANOVA. When there were significant differences, the group means were further compared using Duncan’s multiple range test. *p* < 0.05 was considered to indicate statistical significance. SPSS regression curve estimation and Excel 2010 were used for the fitting function of released ink frequency and released ink weight. Analyses were performed using SPSS version 20.0 (SPSS, Chicago, IL, United States).

## Results

### Cuttlefish Ink Solution Characteristics and Relationship Between Released Ink Frequency and Weight

There was no ink present in the beaker, and an increased height level in the long neck funnel was observed, indicating that the concentration of the ink solution was higher than that of deionized water, and ink solution could not penetrate through the semipermeable membrane (Figure 1A). The light pathway was visible when parallel light passed through the solution, which resulted in the Tyndall effect (Figure 1B). In summary, the cuttlefish ink solution was a homogeneous, stable colloidal solution. The characteristic 320-nm wavelength of the cuttlefish ink solution was obtained by ultraviolet–visible spectrophotometer (UNIC 2800 UV/VIS) full wavelength scanning. Based on the national standard method, the standard curve of concentration was CCI = 89.12 × OD - 0.0299, with *R*^2^ = 0.9999 (Figure 2).

**FIGURE 3:**
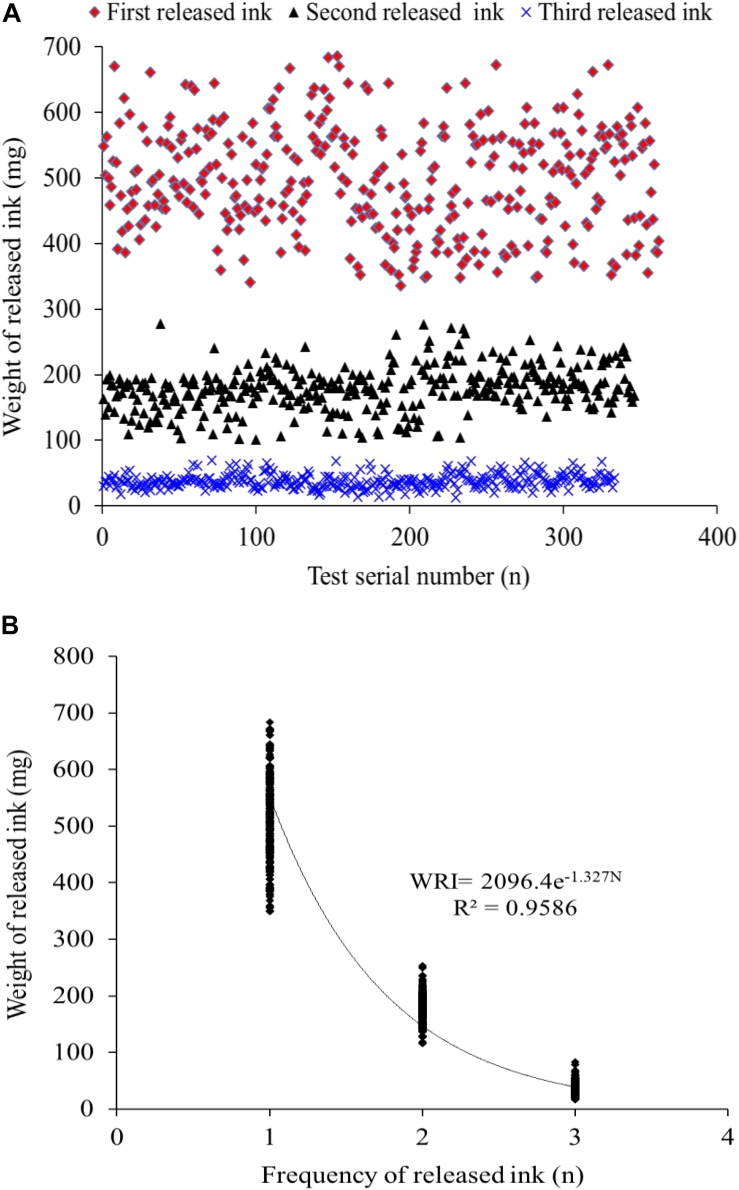
**(A,B)** The distribution of the released ink frequency and the released ink weight and the fitted correlation curve function, respectively (*n* = 333).

The data profile of the first three release ink events is shown in Figure 3A. The weight of each ink release, TRW, and IWIS were analyzed by descriptive statistics (Table 2). As expected, the ink weight of the first ink release event was apparently greater than that of the second event and much greater than that of the third event. According to the calculated IWIS, ∼90% of the ink from the ink sac could be continuously released. The kurtosis and skewness analysis results showed that the ink weight of the first release event and TRW exhibited deviation to the left of the mean, indicating that more data were on the right side of the mean value (André et al., 2014). The fitting function relationship between released ink frequency and weight is shown in Figure 3B. The exponential function is expressed as weight of released ink = 2,096.4 × *e*^–1^.^327^ frequency (*N*), with *R*^2^ = 0.9586.

**TABLE 2:** SPSS descriptive statistics used to analyze the weight of released ink in each release event, total weight of released ink (TRW, mg, *n* = 333), and ink weight in the ink sac (IWIS, mg, *n* = 50) after three ink release events.

**Items**	**Mean**	**SD**	**Max**	**Min**	**Kurtosis**	**Skewness**
First ink release	497.98	80.456	685.99	336.27	–0.5583	0.0191
Second ink release	178.34	33.723	378.03	100.92	0.2962	0.0611
Third ink release	37.91	11.02	69.51	12.33	0.1798	0.5604
TRW	714.23	90.29	963.82	494.16	–1.1166	0.0191
IWIS	764.51	113.98	1108.47	549.84	0.1979	0.1052

### Relationship Between the Released Ink Weight and Mortality at Different Times

**FIGURE 4:**
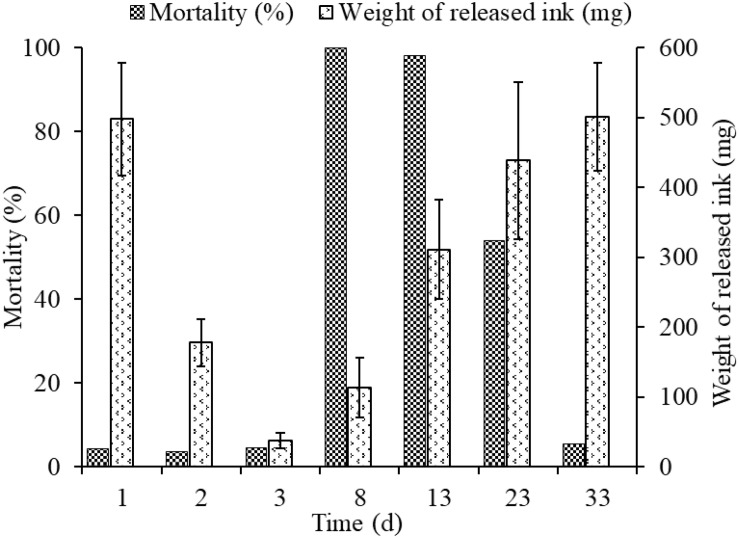
Relationship between weight of released ink and mortality at different times.

The released ink weight dropped sharply throughout the first three inking events, being 497.98 ± 80.4, 178.34 ± 33.7, and 37.91 ± 11.02 mg, in days 1, 2, and 3, respectively, but mortalities in different groups (3.7–4.5%) were low and showed no significant differences among them (Figure 4). The weights of re-released ink events after culture for 5, 10, 20, and 30 days in cement pond were 163.38 ± 42.8, 355.27 ± 70.72, 438.55 ± 112.34, and 501.23 ± 77.34 mg, respectively. However, the mortalities could be as high as 100 and 98% (5 and 10 days of culture, respectively). More importantly, the cuttlefish adapted well to these changes during culture and took as long as 30 days to recover to the initial levels.

### Behavior

**FIGURE 5:**
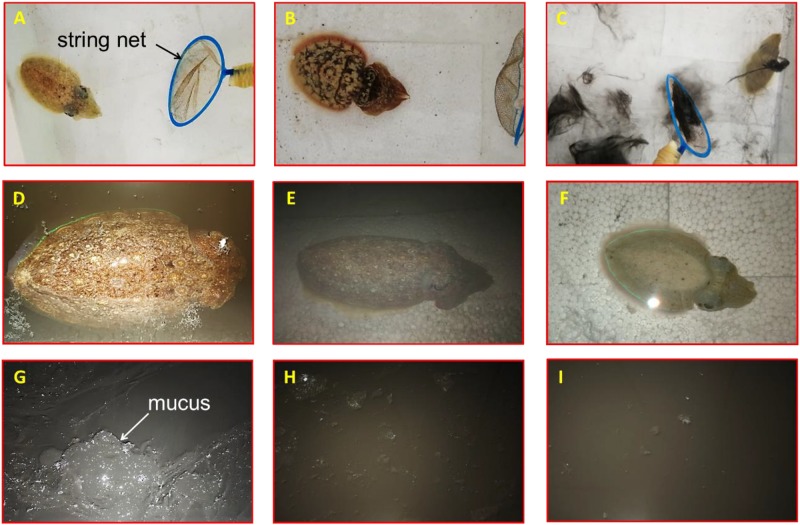
Effect of released ink to behavioral responses of the cuttlefish *Sepia pharaonic*: **(A)** cuttlefish tried to escape at the sight of the predator; **(B)** body transformed to frighten or warn the predator; **(C)** cuttlefish fled covered by inking; **(D)** cuttlefish hovered on the surface; **(E)** cuttlefish sunk to the bottom and hid after continuous jetting/inking; **(F)** body pattern exhibited chromatic, textural, postural, and locomotor features after water condition changed. Properties of released ink: **(G)** dense ink, a gelatinous-like substance with a large amount of mucus, not easy to disperse; **(H)** dense ink, slightly diffuse plume with a small amount of mucus, easy to disperse; **(I)** smoke-like ink, almost no mucus that dispersed quickly.

A comparison of behavioral changes among three inking events is presented in Table 3. The jet frequency of the first ink release event was significantly higher than those of the other events (*p* < 0.05), being 72 ± 11, 46 ± 7, and 28 ± 8, in the first, second, and third event, respectively. The properties of the released ink in the different events were as follows: (1) dense ink, a gelatinous-like substance with a large amount of mucus, which was not easy to disperse (Figure 5G); (2) dense ink, slightly diffuse plume with a small amount of mucus, easy to disperse (Figure 5H); and (3) smoke-like ink, almost no mucus that dispersed quickly (Figure 5I). Behavioral changes and feeding activity were significantly different among inking treatments (Table 3).

**TABLE 3:** Behavioral changes of *Sepia pharaonis* affected by inking treatment.

**Items**	**First ink release**	**Second ink release**	**Third ink release**
Jet frequency	72 ± 11	51 ± 7	28 ± 8
Properties of ink	Dense ink, gelatinous-like,not easy to disperse	Dense ink, slightly diffuse plume, easy to disperse	Smoke-like ink, dispersed quickly
Feeding activity	Food intake decreased, feed utilization about 50–70%	Food intake reduced to half of that of the control group	No difference in food utilization
Locomotor components	Bottom suction, hovering	Bottom suction, hovering	Bottom suction
Chromatic components	Dark brown, dark arms, iridescent blue mantle margin stripe, dark ventral mantle in abdomen	Dark brown, dark arms, wide mantle edge radial bands, dark ventral mantle in abdomen	Pale, iridescent blue mantle margin stripe, pale ventral mantle in abdomen
Textural components	Coarse skin	Coarse skin	Smooth skin
Postural components	Bipod headstand/sitting	Bipod headstand/sitting	Flattened body/sitting
Eye spots	Absent	Absent	Present
Eye ring	Absent	Absent	Present
Dark square	Present	Present	Absent
Fin movement	Absent	Present	Present

The overall responses of a cuttlefish to predators were as follows: first, the cuttlefish tried to escape at the sight of the predator (string net) (no inking) (Figure 5A); second, their body transformed to frighten or warn the predator (Figure 5B); then, the cuttlefish would flee covered by inking after the threat failed (Figure 5C); the cuttlefish hovered on the surface (Figure 5D) or sunk to the bottom and hid after continuous jetting/inking (Figure 5E). The body pattern changes before and after stimuli were classified as follows: the body pattern before stimuli exhibits chromatic (pale, pairs of mantle spots close to the head), textural (smooth skin), postural (flattened), and locomotor (bottom suction) features. The acts of warning or threats to the predator were expressed as a flattening of the body combined with various chromatic components such as eye spots, dark red mantle margin stripe, an iridescent mantle stripe around the fin, and an eye patch in response to a potential threat; the head and arms were flattened with exaggerated arms, the overall form was diamatic flare-like, and the pink fins were also very prominent. The animal fled covered by inking when encountering the predator, and the body pattern exhibited chromatic (pale, white square), textural (smooth skin), postural (streamlined extension), and locomotor (inking/jetting/escaping) features; the cuttlefish hovered on the surface or sunk to the bottom after releasing ink, and the body pattern exhibited chromatic (dark brown), textural (coarse skin), postural (bipod headstand/sitting), and locomotor (bottom suction/hovering) features. After the water condition changed (to fresh seawater), the body pattern exhibited chromatic (pale), textural (smooth skin), postural (flattened/curled arms), and locomotor (bottom suction) features (Figure 5F).

### Enzyme Activity

**FIGURE 6:**
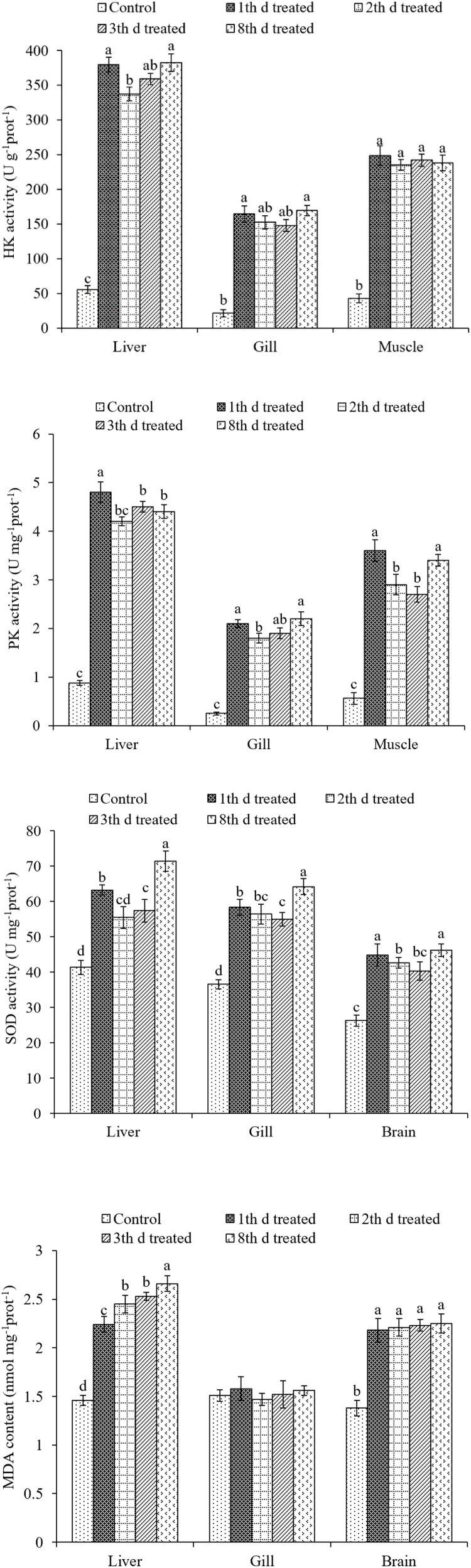
Effects of ink release treatments at different inking events on hexokinase (HK), pyruvate kinase (PK), superoxide dismutase (SOD), and malondialdehyde (MDA) activities in the liver, gill, muscle, and brain tissues of *S. pharaonis*. Data are shown as the mean ± SD. Means within in the same group of bars with different lowercase letters are significantly different (*p* < 0.05).

The effects of treatment on the HK, PK, and SOD activities, as well as MDA content in the liver, gill, muscle, and brain tissues of *S. pharaonis* are shown in Figure 6.

Hexokinase activities were affected by ink release in all tissues (*p* < 0.05). The liver, gill, and muscle tissues showed a rapid increase in the treatment groups, becoming significantly higher than the control group. HK activities in gill and muscle tissues were not significantly different among treatment groups. However, HK activity in liver tissue after the second inking event was lower than on the first and fourth inking events.

The variation in PK activity was similar to that of HK activity. PK activities were higher in the treatment groups compared with the low values in the control group. In liver, gill, and muscle tissues PK activities were much higher after the first inking event than after the following events. PK activities were significantly higher in liver and muscle tissues when compared to those in gill tissue.

Superoxide dismutase activities in the liver, gill, and brain tissues rose with the number of treatments (*p* < 0.05). SOD activities in the liver and gill tissues were significantly higher after the fourth inking event than those after the first, second, and third events. In brain tissues, SOD activity was higher after the first and fourth inking events than after the other two events.

In gill tissue, MDA concentration did not change after treatment. Contrastingly, in the liver tissue, MDA concentration was higher after treatment than that in the control group and increased with each inking event, with the highest value after the fourth event. MDA concentration remained stable after treatment in the brain tissue and was higher than that of the control group.

### Gene Expression

**FIGURE 7:**
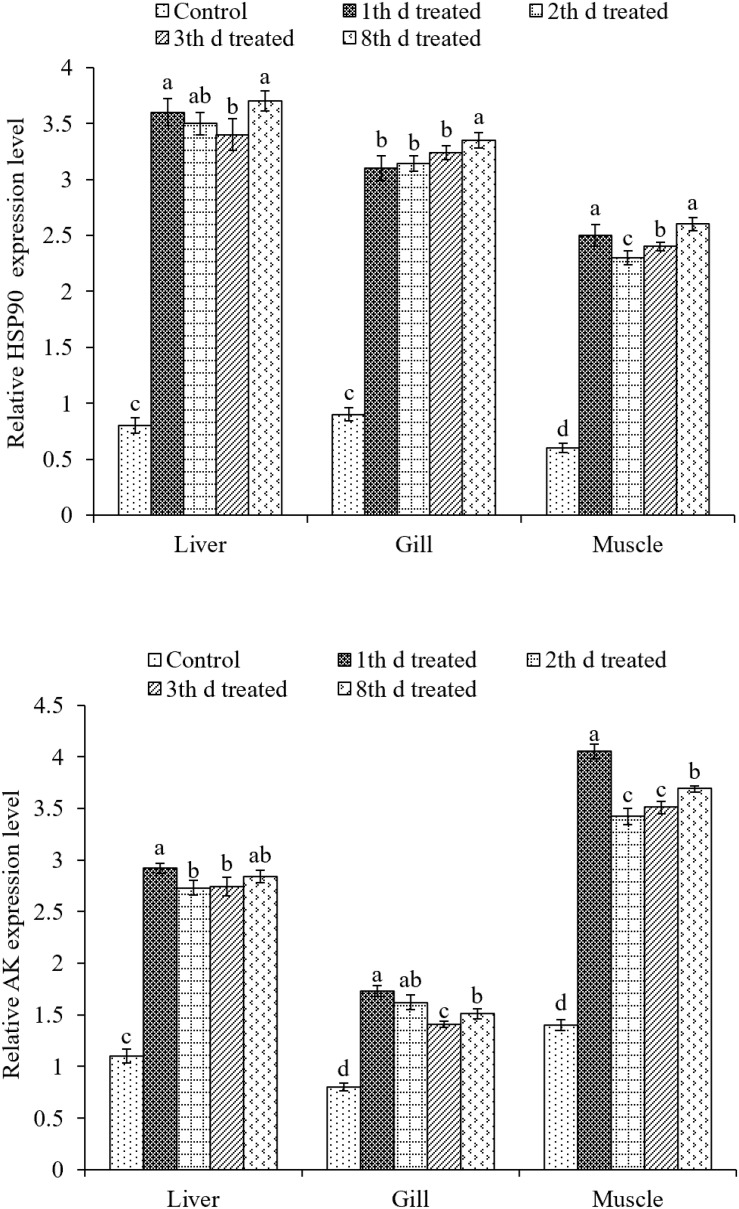
Effects of ink release treatments at different inking events on the relative expression levels of heat shock protein 90 (HSP90) and arginine kinase (AK) in liver, gill, and muscle tissues of *S. pharaonis* at different released ink frequencies. Data are shown as the mean ± SD. Means in the same group of bars with different lowercase letters are significantly different (*p* < 0.05).

The expression of HSP90 was evaluated in liver, gill, and muscle tissues (Figure 7). In liver and muscle tissues, the expression of HSP90 was upregulated after the first and fourth inking events compared to that after the third event. The expression of HSP90 in gill tissues was not different between first, second, and third day of treatment but were much higher at the eighth day of treatment. The highest expression was found in the liver tissues after the fourth inking event.

The expression of AK in liver, gill, and muscle tissues were significantly (*p* < 0.05) upregulated in treatments compared to the control group. AK expression was about two times higher than that of the control group in all treatments. In the liver and muscle, AK expression was upregulated the most after the first inking event. In gill tissues, it was the highest after the first and second inking events.

## Discussion

To evaluate biological and biochemical responses of cuttlefish to ink release, we started with the following two aspects: (1) verified the characteristics of the ink solution and determined the relationship between released ink frequency and weight and (2) clarified the behavioral and physiological effects of continuous inking on cuttlefish.

Predation is a constant risk for most animals. To maximize survival, preys have developed a wide repertoire of defenses from aggressive predators including behavioral displays, physical armor, and toxic chemicals (Bryan et al., 1997; Lenzi-Mattos et al., 2005). In many animal species, body patterns play an important role in predator–prey interactions, such as camouflage, aposematism, crypsis, and visual threats (Endler, 1978; Hay, 2009). Cephalopods such as cuttlefishes, squids, and octopods can not only rapidly change their body color and texture but also exhibit a variety of visually complex appearances. These body patterns include a combination of chromatic, textural, postural, and locomotor components used for camouflage and communication (Hanlon and Messenger, 1988). This study found a series of behavioral changes in cuttlefish *S. pharaonis* while encountering predators. The cuttlefish first displayed defensive behavior against the predator and then fled but without inking or jetting. After increasing the threat proximity, cuttlefish showed a warning or threat to the predator and escapes were accompanied by ink release when an attack was imminent. Finally, the animal sank to the bottom and hid. A total of 53 chromatic and 4 uniform colors, 3 textural, 11 postural, and 9 locomotor components have been previously identified and described in detail for *S. pharaonis* (Nakajima and Ikeda, 2017). In the present study, six chromatic (eyespots, head crown, eye patch, white spots, iridescent ventral mantle, and paired mantle spots close to head), two uniform colors (pale and dark brown), two textural (smooth skin and coarse skin), five postural (flattened, streamlined extension, bipod headstand, sitting and curled arms), and five locomotor (bottom suction, inking, jetting, escaping, and hovering) components were recorded when subjected to potential predators.

To expand our understanding of ink release mechanisms of the cuttlefish *S. pharaonis*, such as continuous ink release capability and behavior, the effect of continuous jetting/inking on the organism, such as biochemical changes and the ink synthesis rate, were investigated. Cuttlefish mortalities from first to third inking events were 4.4% (16/362), 3.7% (13/346), and 4.5% (15/333) (Figure 4), respectively, and the weight of released ink decreased significantly along with the increase in ink release frequency. The exponential function indicated that a cuttlefish has the capability to continuously releasing ink, but the weight of released ink dropped sharply. Results showed that ∼90% of the ink in the ink sac was released (Figure 3A and Table 2). According to the jet frequency, the frequencies of the first three events were 72 ± 11, 51 ± 7, and 28 ± 8, respectively. The higher will be the jet frequency, the more energy will be consumed. Jet frequency decreased with decreasing weight of released ink and the times required to resume swimming after events 1-3 were 6 ± 1 min, 3 ± 1 min, and 3 ± 1 min, respectively. Cuttlefish food intake also decreased after ink release, and it took 2-3 days to recover feeding after three inking events. Gallardo et al. (2017) observed that continuously releasing ink may affect the digestion and utilization of prey in octopods. In our study, cuttlefish required time to “recuperate” to save ink after continuous ink release. After three inking events, 90% of the ink was released, taking ∼30 days for the ink sac to refill. However, the cuttlefish re-release of ink on the 5th and 10th day of recovery resulted in high mortality (98-100%) because continuous burst swimming consumes considerable energy, and over-exploitation probably resulted in serious physiological damage. In nature, the frequency of ink release and the weight of released ink can be adjusted in response to the stress characteristics (e.g., the strength and duration of the predator). Consequently, cuttlefish should be kept away from discomfort environments (such as handling, feeding competition, and rapid temperature and salinity changes) during culture, reducing the possible physiological damage caused by continuous inking. Furthermore, the released ink characteristics were different (i.e., gelatinous mass, slightly diffuse plume, and smoke-like ink), so the defensive effects also varied (Shimek, 1983; Bush and Robison, 2007). Therefore, a short-term ink release could act as a defensive behavior against a predator, but a “powerful” predator forces the animal to continuously release dense ink, which can also cause damage to itself.

Ink release not only requires substantial energy but also consumes considerable oxygen. The liver and gills are the main organs of the cuttlefish, accounting for 35-42 and 18-23% of their visceral mass (*n* = 126), respectively. In addition, liver physiology and biochemical indices can reflect the nutritional and physiological status of aquatic animals (Fontagne et al., 1998; Wang L.N. et al., 2014). Amino acids and carbohydrates are the preferred fuels for cephalopods (Storey and Storey, 1983; Agnisola et al., 1991; Hochachka, 1995; Speers-Roesch et al., 2016), and cuttlefish present well-developed glycolytic capacity in all tissues. After burst swimming, tissue metabolism was similar to that generally seen in starved cuttlefish, and carbohydrates are important fuels in cephalopod muscle (Speers-Roesch et al., 2016). HK and PK enzymes play major roles in the nutritional regulation of glycolytic pathways (Panserat et al., 2001). In this study, HK and PK activities after ink release were significantly higher than those in the control group. The liver and muscle, which fuel locomotion, had high HK and PK activities that geared them toward rapid mobilization of stored glycogen to sustain anaerobic burst swimming. However, the liver relies more on aerobic glucose, as indicated by higher HK and PK activities compared with muscle and gill tissues. Energy was drastically consumed after continuously releasing ink, resulting in death, and the mortality from re-releasing ink was 98-100% on the 5th and 10th day of culture. As the first line of defense, SOD plays pivotal roles in the elimination of reactive oxygen species (ROS) (Kim et al., 2011), and it is thought to protect muscular oxidative stress caused by exercise (Atig et al., 2012). MDA is a peroxidation product of lipids and indirectly reflects the impact degree of ROS on membrane lipid peroxidation (Liu et al., 2013). SOD activity in the treatment groups was higher than that in the control group in the liver, gill, and brain tissues. Especially after the fourth inking event, SOD activity was higher than that from other treatment groups, indicating that continuous ink release could damage the liver and brain tissues. The continuous release of dense ink leads to an increase in the production of active oxygen. To maintain the balance of oxygen free radicals *in vivo*, SOD activity increased to eliminate reactive oxygen radicals, thereby reducing the oxygen free radicals on the biofilm, resulting in an MDA content reduction in the body. Ink release induced the formation of antioxidant enzymes. However, excessive ROS can still cause an increase in MDA content. The increase in MDA levels may be due to the inability of enzymes to prevent extremely high levels of ROS from causing damage to the body (Bagnyukova et al., 2006). Moreover, MDA concentration in the liver tissue significantly increased with each inking event and was higher than that of the control group. In conclusion, liver was affected by continuous ink release.

Heat shock proteins function as molecular chaperones and play essential roles in the immunity of organisms, particularly concerning environmental stress, such as thermal, salinity, and crowding stress (Wang Q.L. et al., 2014; Xu et al., 2014; Galt et al., 2018). In this study, the expression of HSP90 was upregulated in all treatments compared to that in the control group, including liver, gill, and muscle tissues. Interestingly, the expression of HSP90 was more upregulated in liver and gill tissues than in muscle tissues. Increased HSPs in the muscles may facilitate mitochondrial biogenesis and the folding of nuclear gene-encoded proteins into mitochondria. Various studies have revealed that mitochondrial biogenesis is an adaptive response to related stress in the muscles of fish (Tyler and Sidell, 1984; Egginton and Sidell, 1989; Orczewska et al., 2010). Moreover, HSP90 can bind to protein kinases, steroid receptors, actin, tubulin, and other substances in the cells, maintain protein structure, and deliver signals among cells (Pratt, 1997; Csermely et al., 1998). Gao et al. (2008) suggested that the stimulated increase in HSP90 expression level was one of the organisms’ protective approaches against further toxicity.

Previous studies have shown that energy metabolism-related enzymes played an important role in the stress response of invertebrates such as AK (EC 2.7.3.3) (Abe et al., 2007; Yin et al., 2018), which has been found to be closely involved in adaptation to environmental stresses, e.g., pH, salinity, and heavy metal ions, in shrimp, crabs, and cuttlefish (Kinsey and Lee, 2003; Morris et al., 2005; Silvestre et al., 2006; Yin et al., 2018). In this regard, AK in marine invertebrates is distinctively found to be associated with adapting to the environmental disturbances caused by physical and chemical factors (Kinsey and Lee, 2003; Morris et al., 2005). Indeed, AK expression was upregulated in tissues, especially in muscles, which showed higher expression than other tissues. It has been generally recognized that AK plays a pivotal role in ATP buffering in invertebrates under both long-term and extreme conditions, when muscle and nerve cells require immediate and highly fluctuating energy demands via catalyzing Mg^2+^ cofactor-dependent phosphoryl transfer (Shofer et al., 1997; Voncken et al., 2013). Furthermore, expression screening and annotation of the ink sac cDNA library indicated that the main related unigenes were related to carbohydrate, amino acid, and energy metabolism, as well as cell motility (Song et al., 2012).

In summary, the study showed that cuttlefish can continuously release ink within a short period, releasing ∼90% of the ink from the ink sac, but re-releasing ink during the recovery period was prone to cause exhaustion and death. Moreover, the cuttlefish adapted well to these changes during culture and took as long as 30 days to recover. Profiles of biochemical indicators indicated that continuously releasing ink damaged tissues, especially the liver tissue. Understanding the stress response to stimulus of cuttlefish is critical for optimizing production and maintaining health and welfare. Although cuttlefish can continuously release ink in to defend against predators, this defensive behavior can result in physiological damage and death. However, their intrinsic link is still not clearly understood. Therefore, further study may focus on metabolomics and the relationship between metabolites and physiological and pathological changes after ink release.

## Data Availability Statement

All datasets generated for this study are included in the article/supplementary material.

## Ethics Statement

All procedures with live animals included in this study have been approved by the Animal Research Ethics Committee of the Chinese Academy of Fishery Sciences. Research on live cephalopods is now regulated in the Member States of the European Union by the Directive 2010/63/EU. The authors are aware of the general principles stated by the Directive for the use of live cephalopod mollusks in scientific research as pointed out in several studies (Smith et al., 2013; Fiorito et al., 2015).

## Author Contributions

MJ, WS, and XJ conceived and designed the experiments. CZ and RY ran the experiments. JL and RP sampled animals and performed RNA extractions. MJ and WS wrote the manuscript. QH revised the manuscript. XJ provided funding and helped in discussing the results.

## Conflict of Interest

The authors declare that the research was conducted in the absence of any commercial or financial relationships that could be construed as a potential conflict of interest.

## Footnotes

1 www.ysi.com

2 http://www.njjcbio.com/
